# Magnetic Porous Molecularly Imprinted Polymers Based on Surface Precipitation Polymerization and Mesoporous SiO_2_ Layer as Sacrificial Support for Efficient and Selective Extraction and Determination of Chlorogenic Acid in Duzhong Brick Tea

**DOI:** 10.3390/molecules23071554

**Published:** 2018-06-27

**Authors:** Mijun Peng, Huan Li, Ruiqing Long, Shuyun Shi, Hanjun Zhou, Shuping Yang

**Affiliations:** 1 Provincial Public Laboratory of Analysis and Testing Technology, Guangdong Institute of Analysis, Guangzhou 510070, China; pengmj163@163.com; 2 College of Chemistry and Chemical Engineering, Central South University, Changsha 410083, China; huxcsu@126.com (H.L); cphacm@126.com (R.L.); 3 National & Local United Engineering laboratory of Integrative Utilization Technology of Eucommia ulmoides, Jishou University, Jishou 416000, China; 4 School of Mathematics and Statistics, Central South University, Changsha 410083, China; zhou1125870206@outlook.com

**Keywords:** surface precipitation polymerization, mesoporous SiO_2_, selective extraction, chlorogenic acid, Duzhong brick tea

## Abstract

Magnetic porous molecularly imprinted polymers (MPMIPs) for rapid and efficient selective recognition of chlorogenic acid (CGA) were effectively prepared based on surface precipitation polymerization using CGA as template, 4-vinylpyridine (4-VP) as functional monomer, and mesoporous SiO_2_ (mSiO_2_) layer as sacrificial support. A computational simulation by evaluation of electronic binding energy is used to optimize the stoichiometric ratio between CGA and 4-VP (1:5), which reduced the duration of laboratory trials. The porous MIP shell and the rid of solid MIPs by magnet gave MPMIPs high binding capacity (42.22 mg/g) and fast kinetic binding (35 min). Adsorption behavior between CGA and MPMIPs followed Langmuir equation and pseudo-first-order reaction kinetics. Furthermore, the obtained MPMIPs as solid phase adsorbents coupled with high performance liquid chromatography (HPLC) were employed for selective extraction and determination of CGA (2.93 ± 0.11 mg/g) in Duzhong brick tea. The recoveries from 91.8% to 104.2%, and the limit of detection (LOD) at 0.8 μg/mL were obtained. The linear range (2.0–150.0 μg/mL) was wide with *R*^2^ > 0.999. Overall, this study provided an efficient approach for fabrication of well-constructed MPMIPs for fast and selective recognition and determination of CGA from complex samples.

## 1. Introduction

Quantification of trace compounds in complex matrices is always a big analytical challenge. In order to extract target compounds and get rid of interferences (as well as sample matrix) for accurate and sensitive analysis, extraction methods (e.g. liquid–liquid extraction, solid-phase extraction) have been emerged [1,2,3]. Solid-phase extraction gained wider acceptance because of its more efficient separation process, use of greener and less quantities of organic solvents, better recovery, and higher preconcentration factor than liquid–liquid extraction [4]. Up to date, different types of sorbents have been developed for solid-phase extraction, such as C_8_/C_18_-bonded silica [4], carbon materials [5], polymeric ionic liquids [6], metal–organic frameworks [7], mesoporous or nanoporous silica [8], molecularly imprinted polymers (MIPs) [9,10,11,12], etc. Among them, MIPs contained specific recognition sites for target compounds, therefore, MIPs had high affinity and selectivity [9,10,11,12]. Nowadays, MIPs have been widely investigated and applied as solid-phase extraction sorbents.

In the preparation of MIPs, bulk polymerization, suspension polymerization, and precipitation polymerization were the conventional polymerization methods [13]. One of the main problems was their need to centrifuge or filtrate for isolation from solution. Gratifyingly, magnetic MIPs have been developed [11,14,15,16]. Magnetic MIPs could be separated under an external magnet. At the same time, surface recognition sites made adsorption and desorption rapid. It was obvious that larger surface area of solid core resulted in high densities of recognition sites on the surface, and then, the higher binding capacity. Therefore, magnetic mesoporous SiO_2_ (Fe_3_O_4_@mSiO_2_) was an attractive alternative. Protocatechuic acid imprinted polymers on Fe_3_O_4_@mSiO_2_ had adsorption capacity 2.3 times that on Fe_3_O_4_@SiO_2_ [17]. It was noted that the mesoporous structure of mSiO_2_ made MIPs synthetic steps simple and economical (i.e. not using expensive 3-(methacryloxy) propyl trimethoxysilane as functional material to modify the surface of SiO_2_) [15]. Satisfactorily, solid MIPs synthesized outside mSiO_2_ could be removed magnetically. Furthermore, mSiO_2_ as sacrificial support exited significant benefits: (1) porous structure for fast mass transfer; (2) removal of solid core without any recognition sites for increased binding capacity per unit mass [15]. As a result, MPMIPs as selective sorbents presented advantages.

Recognition properties of MIPs were affected by the type of functional monomer and the molar ratio of functional monomer and template. Optimized experiments by trial and error were tedious, while computational simulations were more economical and less time-consuming than experiments. Thus, computer-aided simulations provided a useful prospective in design and synthesis of MIPs [18,19,20]. In the preparation of MIPs, template–functional monomer complex was firstly formed by hydrogen bond. Then, the binding energy (ΔE) between template and functional monomer was a guideline for the selection of proper functional monomer. Generally, the bigger value of ΔE, the stronger interaction between template and functional monomer, and then the higher selectivity of the MIPs. Semi-empirical quantum mechanical methods (PM3) and density functional theory (DFT) were precise and efficient algorithms to calculate hydrogen bond energy, which were then widely used in theoretical and computational chemistry to calculate ΔE for MIPs design.

Chlorogenic acid (CGA), with various pharmacological activities (e.g. antioxidant, anti-inflammatory, antibacterial, antiviral, anti-hypertension, anti-obesity, antipyretic, hepatoprotective, neuroprotective) [21], is widely distributed in plant-derived materials. Therefore, selective extraction of CGA from complex matrices was of great importance before analytical procedures. Recently, surface CGA imprinted MIPs on magnetic Fe_3_O_4_, hollow fiber, nano-TiO_2_, and graphene–carbon nanotube, were fabricated [22,23,24,25]. In order to form high-density recognition site, Zhao and coworkers increased the number of amino groups on the surface of magnetic Fe_3_O_4_ to immobilize more CGA [24]. Ji et al. optimized the type of functional monomer and crosslinker, and the molar ratio of template/functional monomer/crosslinker to increase binding capacity [26]. However, to our best knowledge, no paper has reported the preparation of MPMIPs for effective and selective extraction of CGA from complex matrices.

Duzhong brick tea, as a new functional tea, was available on the Chinese market for specified health uses; for weight management, obesity prevention, blood pressure reduction, as well as an antioxidant agent. However, to our best knowledge, its components and the content of CGA were unclear. Then, in the present study, with the theoretical calculation results, MPMIPs, using Fe_3_O_4_ as magnetic core, and porous CGA imprinted MIPs as shell, were prepared for the first time. The characterization, adsorption isotherms/kinetics, and adsorption specificity of MPMIPs and magnetic porous non-molecularly imprinted polymers (MPNIPs) were investigated. Then MPMIP-based solid phase extraction coupled with high performance liquid chromatography (HPLC) were developed and validated for selective determination of CGA in Duzhong brick tea.

## 2. Results and Discussion

### 2.1. Molecular Simulation and Calculation of Energies

Strong interaction between template and functional monomer is the key point in selective recognition of MIPs. Theoretical calculations have become popular procedures during functional monomer screening and the molar ratio optimization of functional monomer and template for preparation of MIPs, because of their time and cost saving by comparison with experiments [18,19,20]. Noncovalent binding, especially for hydrogen bonding, commonly existed between template and functional monomer in the formation of recognition sites. PM3 and DFT were generally preferred methods for calculation of hydrogen bonding energy [27]. Calculation of the binding energy (*ΔE*) was based on the following formula:

ΔE = E_complex_ − (E_CGA_ + nE_4 − VP_) (1)

where E_complex_, E_CGA_, and E_4−VP_ represent the energy of CGA–4-VP complex, CGA, and 4-VP, respectively, and n is the number of 4-VP in CGA–4-VP complex.

Li et al. compared the binding energy of complexes between CGA and different functional monomers (acrylamide, acrylic acid, methacrylic acid, 2-vinyl pyridine, and 4-VP), and results indicated that CGA interacted most strongly with 4-VP [20]. Ji et al. also found that MIPs for CGA using 4-VP as functional monomer had higher binding capacity and imprinting factor than those using methacrylic acid as functional monomer [26]. Then, 4-VP was selected as the most appropriate functional monomer for preparation of CGA imprinted polymers. The binding energies of CGA–4-VP complexes were calculated. As shown in Table 1, lowest binding energy happened for CGA–4-VP complex with a molar ratio at 1:5 in both PM3 and DFT simulations. As a result, the molar ratio of 1:5 for CGA–4-VP complex was the most beneficial to prepare highly selective MIPs.

### 2.2. Characterizations

**Figure 1:**
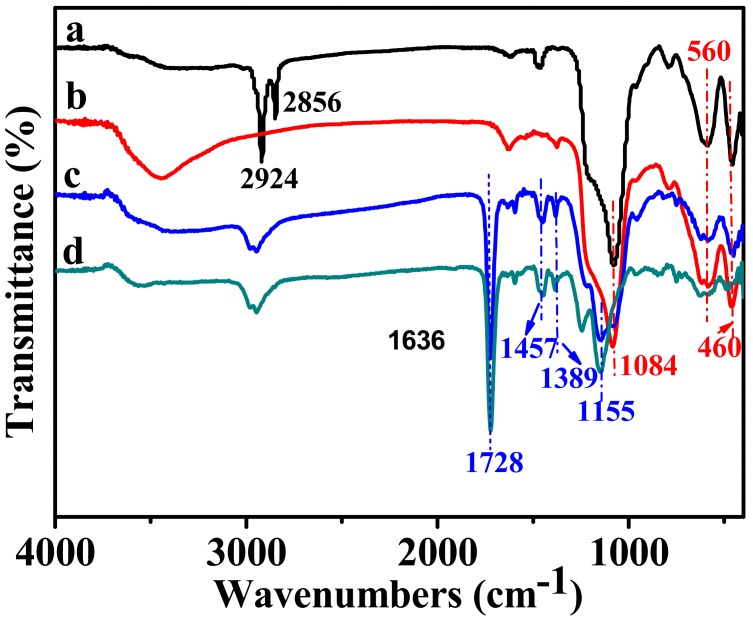
FT-IR spectra of Fe_3_O_4_@mSiO_2_/CTAB (**a**), Fe_3_O_4_@mSiO_2_ (**b**), Fe_3_O_4_@mSiO_2_@MIPs (**c**), and magnetic porous molecularly imprinted polymers (MPMIPs) (**d**).

The FT-IR spectra of Fe_3_O_4_@mSiO_2_/CTAB, Fe_3_O_4_@mSiO_2_, Fe_3_O_4_@mSiO_2_@MIPs, and MPMIPs were displayed in Figure 1. The adsorption band at 560 cm^−1^ in Figure 1a could be assigned to Fe–O stretching vibration. Two characteristic adsorption bands at 1084 and 460 cm^−1^ corresponded to the Si–O asymmetric stretching and bending vibrations, respectively. In Figure 1a, C–H stretching vibration peaks at 2924 and 2856 cm^−1^ were ascribed to CTAB, which disappeared in Figure 1b with the complete removal of CTAB. Figure 1c showed the C=O, C=C, C–O–C, and C=N stretching bands at 1728, 1457, 1155, and 1389 cm^−1^, which implied the successful polymerization. After reaction with NaOH, the characteristic vibrations for SiO_2_ disappeared in Figure 1d. The results indicated the successful preparation of MPMIPs. Moreover, the disappearance of broad reflection of well-ordered mesoporous silica in XRD pattern (2*θ* = 20–25, Figure S1) for MPMIPs demonstrated the complete removal of SiO_2_. As a result, the MIPs content increased from 54.42% in Fe_3_O_4_@mSiO_2_@MIPs to 73.11% in MPMIPs (Figure S2).

**Figure 2:**
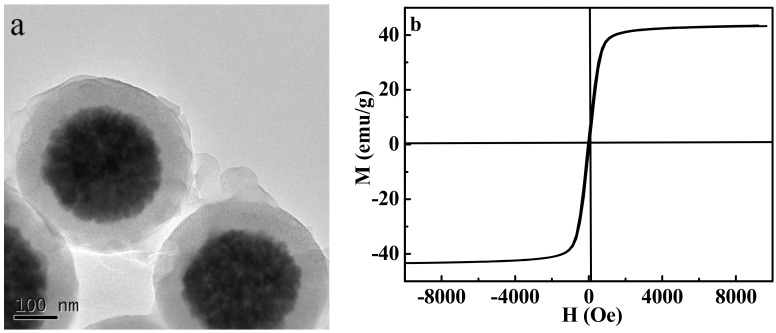
TEM image (**a**) and hysteresis loop (**b**) for MPMIPs.

Figure 2a depicted the TEM image of MPMIPs with core–shell structure. The mean diameter of MPMIPs was about 400 nm, and the thickness of imprinted polymer layer was about 60 nm. Figure 2b showed the hysteresis loop of MPMIPs, and the magnetic saturation was about 43.51 emu/g at the field of 10 KOe, which indicated that MPMIPs could be quickly separated by an external magnet. The porous structure of MIPs layer could be detected by nitrogen adsorption–desorption experiment (data not shown), which indicated that MPMIPs had a pore approaching micropore (2.94 nm, the wall thickness of MCM-48) [14].

### 2.3. Adsorption of CA on MPMIPs/MPNIPs

#### 2.3.1. Adsorption Kinetics

The binding kinetics were investigated because they defined the efficiency of adsorption. The adsorption capacity *Q_t_* (mg/g) at contact time *t* was calculated as

*Q_t_* = (*C_0_* − *C_t_*) × *V*/*m* (2)

where *C_0_* (mg/mL) and *C_t_* (mg/mL) were the initial CGA concentration, and CGA concentration at contact time *t*, respectively, and *V* (mL) was the volume of CGA solution, and *m* was the mass of MPMIPs and MPNIPs (g).

**Figure 3:**
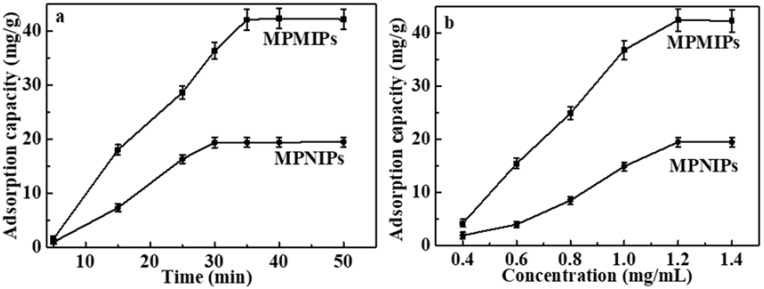
(**a**) Kinetic adsorption curves of MPMIPs/magnetic porous non-molecularly imprinted polymers (MPNIPs) by addition of 100.0 mg polymers in CGA solution (1.2 mg/mL) for 0–50 min; (**b**) Equilibrium adsorption curves of MPMIPs/MPNIPs by addition of 100.0 mg polymers in CGA solution (0.4–1.4 mg/mL) for 35 min (*n* = 3).

Figure 3a displayed the kinetic curves of CGA adsorption on MPMIPs/MPNIPs. CGA adsorption increased with the increment of adsorption time, and reached adsorption equilibrium at about 35 min, suggesting that the adsorption was a fast process. Therefore, porous structure in MPMIPs/MPNIPs promoted the diffusion of CGA to recognition sites, inducing a much faster adsorption rate.

Pseudo-first-order and pseudo-second-order models were selected to analyze the kinetic data.

(3) $$\mathrm{Pseudo}-\mathrm{first}-\mathrm{order}:\;\ln(Q_{e}-Q_{t})=\ln Q_{e}-K_{1}t$$

(4) $$\mathrm{Pseudo}-\mathrm{second}-\mathrm{order}:\;\frac{t}{Q_{t}}=\frac{t}{Q_{e}}+\frac{1}{Q_{e}^{2}K_{2}}$$

where *K*_1_ (min^−1^) and *K*_2_ (g/(mg·min)) represent the pseudo-first-order and pseudo-second-order rate constants, respectively. As can be seen from Table S1, the pseudo-first-order rate model was better to fit the kinetic data. The results suggested the diffusion-controlled process between CGA and MPMIPs.

#### 2.3.2. Adsorption Isotherms

Static adsorption was carried out to evaluate the imprinting effect. The equilibrium concentrations of CGA, *Q_e_* (mg/g), were calculated based on the following equation:

*Q_e_* = (*C_0_* − *C_e_*) × *V*/*m* (5)

where *C_0_* and *Ce* (mg/mL) represented the initial and equilibrium concentrations of CGA.

The equilibrium adsorption of CGA on MPMIPs/MPNIPs were measured in different initial concentrations, and the results were displayed in Figure 3b. The equilibrium adsorption capacities increased with the increase of initial concentrations, and the adsorption saturated when the concentration of CGA reached 1.2 mg/mL. The maximum equilibrium capacity of CGA on MPMIPs was predicted to be 42.22 mg/g, 2.17 times that of MPNIPs (19.42 mg/g), because of the existence of specific imprinting effect. Although magnetic core had no beneficial effect on binding capacity, it was higher than that on hollow MIPs (26.95 mg/g) [25]. That was because of the removal of co-synthesized solid MIPs from MPMIPs by magnet.

The Langmuir and Freundlich models were selected to estimate the binding properties of MPMIPs and MPNIPs.

(6) $$\mathrm{Langmuir}\;\mathrm{equation}:\;1/Q_{e}=1/(K_{L}C_{e}Q_{m})+1/Q_{m}$$

(7) $$\mathrm{Freundlich}\;\mathrm{equation}:\;\log Q_{e}=(\log Q_{e})/n+\log K_{F}$$

where *Q_m_* (mg/g) represents the maximum adsorption capacity, *K_L_* (mL/mg) is the Langmuir adsorption coefficient, and *n* and *K_F_* are Freundlich constants. As summarized in Table S2, Langmuir equation was more appropriate for fitting the isotherm adsorption than Freundlich equation since *R^2^* > 0.97, which implied that the recognition sites were uniformly distributed in a monolayer on the adsorbent surface.

#### 2.3.3. Adsorption Selectivity

**Figure 4:**
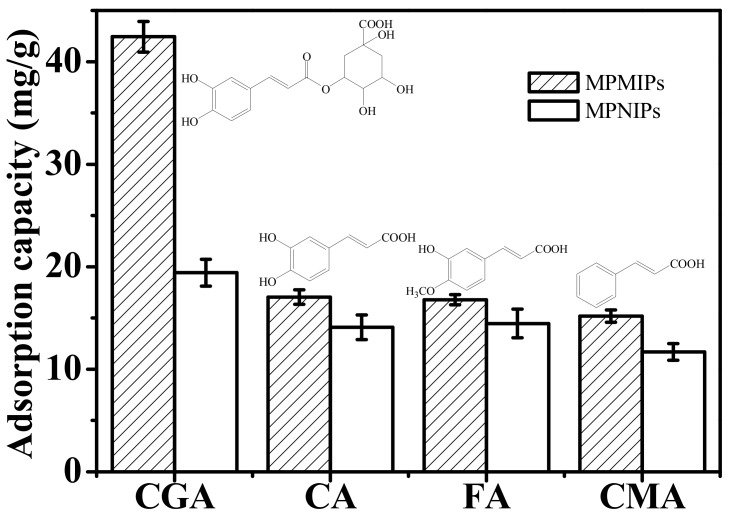
Adsorption capacities of 1.2 mg/mL of CGA, CA, FA, and CMA on MPMIPs/MPNIPs for 35 min (*n* = 3).

Three analogues of CGA, caffeic acid (CA), ferulic acid (FA), and cinnamic acid (CMA), were selected to investigate the selectivity of MPMIPs and MPNIPs. As shown in Figure 4, MPMIPs contained the highest adsorption capacity for CGA, which was about 2.49, 2.53, and 2.80 times that for CA, FA, and CMA. Molecular recognition ability was related to the similarity between CGA and MPMIPs in size, shape, and functional groups. CA with shortened side chain length resulted in lower binding capacity. The adsorption capacities of FA and CMA were lower than that of CGA, which might be caused by their different molecular size, resulted from the different numbers of phenolic hydroxyl or methoxyl group. The binding-selectivity coefficients for CGA, CA, FA, and CMA were calculated and displayed in Table S3; *K_d_* (distribution coefficient) values of five analytes on MPMIPs were larger than those of MPNIPs, and *K_d_* value of CGA was greater than those for three analogues, which indicated the binding selectivity of CGA on MPMIPs. The *K′* (relative selectivity coefficient) values were larger than 1, which showed that imprinting effect played an essential role in the high selectivity of MPMIPs for CGA. In a word, MPMIPs could be applied as a selective sorbent for CGA from real complex matrices.

### 2.4. Analysis of CGA in Duzhong Brick Tea

Under optimized conditions, a calibration curve with *R^2^* >0.999 was established for CGA in the concentration range of 2.0–150.0 μg/mL. The calculated regression equation was *Y* = 56.10*X* − 23.02, where *Y* and *X* were the peak areas and CGA concentrations, respectively. The LOD (limit of detection) was 0.8 μg/mL (*S/N* = 3) and the LOQ (limit of quantification) was 1.5 μg/mL (*S/N* = 10). To further verify the accuracy of this method, the recovery tests were performed by spiking different concentrations of CGA standard solutions into Duzhong brick tea, then extracted by MPMIPs and recoveries from 91.8% to 104.2% with RSD values less than 10.0% were achieved. The RSD% for intraday precision (2.5–5.8%) and interday precision (4.5–9.2%) were found, which indicated high reproducibility. 

**Figure 5:**
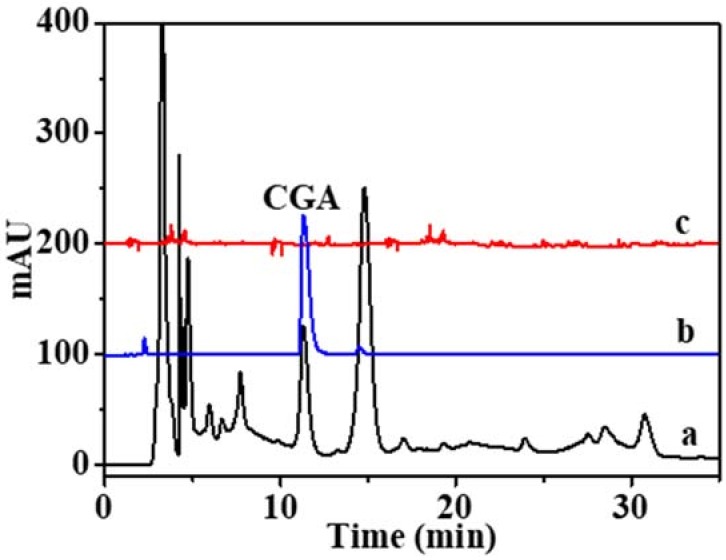
HPLC chromatograms of Duzhong brick tea extract. (**a**) The chromatogram of initial Duzhong brick tea extract; (**b**) The chromatogram of elution of CGA after extracted with MPMIPs; (**c**) with MPNIPs.

As illustrated in Figure 5, MPMIPs showed a good selective separation of CGA from other compounds. Finally, CGA in Duzhong brick tea was estimated at concentrations of 2.93 ± 0.11 mg/g.

## 3. Experimental

### 3.1. Chemicals and Reagents 

Iron(III) chloride hexahydrate (FeCl_3_·6H_2_O) and 2,2-azobis (isobutyronitrile) (AIBN) were purchased from Kemiou Chemical Reagent Co., Ltd (Tianjin, China). Polyethylene glycol 6000 (PEG 6000), cetyltrimethyl ammonium bromide (CTAB), tetraethyl orthosilicate (TEOS), sodium hydroxide (NaOH), anhydrous acetonitrile, methanol, ethanol, sodium acetate, ethylene glycol and HPLC grade acetonitrile were obtained from Sinopharm Chemical Reagent Co., Ltd (Shanghai, China). Ethylene glycol dimethacrylate (EGDMA) was supplied by Shaen Chemical Technology Co., Ltd (Shanghai, China). CA and CGA were bought from Xiya Reagent Co., Ltd. (Linshu City, Shandong, China). FA, CMA, and 4-vinyl pyridine (4−VP) were afforded by Aladdin Industrial Corporation (Shanghai, China).

### 3.2. Molecular Simulation and Calculation of Energies

The theoretical calculations were on the high-performance computing platform of Central South University, China. Firstly, geometries of CGA and 4-VP were optimized. Secondly, CGA–4-VP interaction was optimized, and their minimal interaction energies in different proportions were calculated by Gaussian 03 software (Gaussian, Inc., Wallingford, CT, USA). In this process, the bonding length beyond the range of hydrogen bond was excluded. PM3 and DFT were applied in the molecular simulation to evaluate the value of ΔE [18,19,20].

### 3.3. Preparation of MPMIPs

Magnetic Fe_3_O_4_ nanoparticles were prepared as our previously reported [14]: FeCl_3_·6H_2_O (1.40 g), sodium acetate (3.60 g) and polyethylene glycol (1.00 g) were dissolved in ethylene glycol (40 mL) and stirred at 30 °C for 60 min. After that, the mixtures were sealed in a Teflon-lined stainless steel autoclave at 200 °C for 8 h; the resulting Fe_3_O_4_ nanoparticles were filtrated, and rinsed by water. After that, Fe_3_O_4_@mSiO_2_ nanoparticles were fabricated through surfactant based sol–gel approach: Fe_3_O_4_ nanoparticles (50.0 mg) and CTAB (500.0 mg) were poured in NaOH aqueous solution (1.0 mM, 450.0 mL) and mechanically stirred at 60 °C for 40 min; after that, TEOS/ethanol (1/4, *v*/*v*) solution (2.5 mL) was added, and the reaction was carried out at room temperature for 12 h to achieve Fe_3_O_4_@CTAB/SiO_2_ nanoparticles; finally, Fe_3_O_4_@mSiO_2_ nanoparticles were collected magnetically after being refluxed at 80 °C for 24 h to remove CTAB.

Subsequently, MPMIPs were synthesized as follows. Fe_3_O_4_@mSiO_2_ (80 mg), CGA (0.25 mmol), 4-VP (1.25 mmol), EGDMA (5.0 mmol) and AIBN (0.15 g) were mixed in acetonitrile (30.0 mL) solution, and mixtures were stirred at 60 °C for 24 h under nitrogen. In this reaction, Fe_3_O_4_@mSiO_2_, CGA, 4-VP, EGDMA, and acetonitrile were solid support, template, functional monomer, crosslinker, and porogen, respectively. Fe_3_O_4_@mSiO_2_@MIPs nanoparticles were collected magnetically, and then ultrasonicated in NaOH solution (5%, 20 mL) [14]. Finally, MPMIPs were achieved after being washed with methanol and dried under vacuum at 60 °C to constant weight. MPNIPs were synthesized using the same procedures with the lack of template CGA.

### 3.4. Characterization 

Fourier transform infrared (FT-IR) spectra of the nanoparticles were recorded on a Nicolet 670 system (Thermo Scientific Nicolet Instrument Corporation, Woodland, CA, USA) with the wavenumber ranged from 400 to 4000 cm^−1^. The morphology of MPMIPs was determined on a transmission electron microscopy (TEM, JEM–2100F, JEOL, Tokyo, Japan). Brunauer–Emmett–Teller (BET) surface area and pore size distribution were determined by N_2_ adsorption–desorption isotherms in a Micromeritics ASAP 2020 device (Micromeritics, Norcross GA, USA). Magnetization curves were observed on a vibrating sample magnetometer (VSM7407, Lake Shore Cryotronics, Inc., Westerville, OH, USA). Wide-angle X-ray diffraction (XRD) patterns were operated on X-ray diffractometer (Rigaku RINT 2500, Rigaku Corporation, Japan). Thermogravimetric analysis was carried out by TGA SDTQ600 device (TA Instruments, New Castle, DE, USA). 

HPLC analysis was carried out using an Agilent 1260 HPLC system with UV detector at 254 nm (Agilent Technologies, Santa Clara, CA, USA). An Agilent ZORBAX SB-C_18_ chromatographic column (250 mm × 4.6 mm i.d., 5 μm, Agilent, Santa Clara, CA) was selected for separation, using 0.4% acetic acid (A)/acetonitrile (B) as the mobile phase (0–10 min, 12% B; 10–16 min, 12–18% B; 16–35 min, 18% B) with a flow rate at 0.8 mL/min. Each sample (20 μL) was injected into HPLC after passing through a 0.22 μm polytetrafluoroethylene syringe filter.

### 3.5. Adsorption Experiments

The kinetic adsorption of MPMIPs/MPNIPs was performed in a thermostatic oscillator. MPMIPs/MPNIPs (100.0 mg) were mixed with CGA solution (1.2 mg/mL, 50.0 mL) in a cone-shaped flask, and then the flask was shaken at 25 °C and 200 rpm until equilibrium. In the process, solution (0.1 mL) was withdrawn at a preset interval, and the concentration of CGA was determined by HPLC.

The equilibrium adsorption of MPMIPs/MPNIPs was evaluated as follows. MPMIPs/MPNIPs (100.0 mg) were mixed with CGA solution (50.0 mL) in difference initial concentrations (from 0.4 to 1.4 mg/mL), and then mixtures were shaken at 25 °C and 200 rpm until equilibrium. After that, the supernatants were measured by HPLC. 

The selectivity adsorption of MPMIPs/MPNIPs was investigated using CGA and its three structurally related compounds (CA, FA, and CMA). MPMIPs/MPNIPs (100.0 mg) were separately incubated with CGA, CA, FA, and CMA solution (1.2 mg/mL, 50.0 mL) at 25 °C. After equilibrium, MPMIPs/MPNIPs were magnetically isolated, and the concentrations of CGA, CA, CMA, and FA were determined by HPLC.

### 3.6. Analysis of CGA in Duzhong Brick Tea

Duzhong brick tea was provided by Zhangjiajie Chakunyuan Biotechnology Development Co., Ltd. (Zhangjiajie, Hunan, China). CA brick tea was ground, sieved, and filtered through a 40-mesh screen. The powders (4.0 g) were separately extracted with 75% ethanol (40 mL) at 85 °C for 3 h, and the extraction procedures were repeated three times. After filtration, the combined extract solutions were concentrated to dryness under reduced pressure to yield Duzhong brick tea extract (0.8 g).

Duzhong brick tea extract solution (10.0 mg/mL in 5.0 mL of acetonitrile) was prepared, and then MPMIPs/MPNIPs (30.0 mg) were added, and the mixtures were shaken at 25 °C and 200 rpm for 35 min in a thermostatic oscillator. Then, MPMIPs/MPNIPs were separated by an external magnet, and washed by acetonitrile. Finally, CGA was released from MPMIPs/MPNIPs by methanol/acetic acid (9:1, *v/v*, 3.0 mL), and CGA solution was magnetically separated. The resultant solution was for the subsequent HPLC analysis [28].

### 3.7. Statistical Analysis

All experiments were performed in a minimum of triplicate, and data were presented as mean ± standard deviation. Graphics were drawn using software OriginPro (OriginLab Corporation, Northampton, MA, USA).

## 4. Conclusions

MPMIPs were successfully fabricated for selective adsorption of CGA, using Fe_3_O_4_ as magnetic core, CA and 4-VP as template and functional monomer for surface precipitation polymerization, and mSiO_2_ layer as sacrificial support. Theoretical calculations were performed for optimization of the mole ratio of CGA and 4-VP. The MPMIPs adsorption behavior demonstrated that MPMIPs had easy separation, fast binding kinetics, high adsorption capacity, and good selectivity to CGA. Subsequently, selective adsorption of CGA from Duzhong brick tea with high recovery rates and then the quantification of CGA by HPLC were successfully performed, demonstrating that the synthesized MPMIPs could rapidly, efficiently and selectively recognize CGA from complex samples.

## Figures and Tables

**Table 1:** Binding energies of chlorogenic acid (CGA) with 4-vinylpyridine (4-VP) in different molar ratio.

CA/4-VP	Δ*E*	
	PM3 (kJ/mol)	DFT (kJ/mol)
1:1	−18.38	−43.06
1:2	−34.13	−78.76
1:3	−55.14	−110.27
1:4	−68.26	−118.15
1:5	−91.89	−168.03
1:6	−70.89	−131.22

## Supplementary Materials

The following are available online. Figure S1: Wide-angle XRD patterns of Fe3O4@mSiO2@MIPs (a) and MPMIPs (b), Figure S2: TGA curve of Fe3O4@mSiO2@MIPs (a), MPMIPs (b), Table S1: Kinetic constants for the pseudo-first-order and pseudo-second-order rate equations, Table S2: Adsorption isotherm constants for Langmuir and Freundlich equations, Table S3: The selectivity parameters of MPMIPs and MPNIPs.
