# Supplementary material for: Magnetic Porous Molecularly Imprinted Polymers Based on Surface Precipitation Polymerization and Mesoporous SiO2 Layer as Sacrificial Support for Efficient and Selective Extraction and Determination of Chlorogenic Acid in Duzhong Brick Tea
Source: Molecules. 2018 Jun 27;23(7):1554. doi: 10.3390/molecules23071554 (PMC6099399; doi:10.3390/molecules23071554)

Table S1

Kinetic constants for the pseudo-first-order and pseudo-second-order rate equations.

	Pseudo-first-order			Pseudo-second-order		
	K_1(min^{-1})	Q_m(mg/g)	R^2	K_2(g·mg-1)	Q_m(mg/g)	R^2
MPMIPs	0.02	66.34	0.929	0.00049	30.58	0.346
MPNIPs	0.03	27.31	0.880	0.00050	23.64	0.280

Table S2

Adsorption isotherm constants for Langmuir and Freundlich equations.

	Langmuir			Freundlich		
	K_L(mL/mg)	Q_m(mg/g)	R^2	n	K_F	R^2
MPMIPs	2.94	47.86	0.986	0.53	34.77	0.908
MPNIPs	1.82	22.82	0.977	0.49	13.00	0.973

Table S3

The selectivity parameters of MPMIPs and MPNIPs.

		CGA	CA	FA	CMA
MPMIPs	Q (mg/g)	42.44	17.05	16.79	15.18
	K_d (mL/g)	38.06	14.62	14.39	12.98
	K		2.60	2.64	2.93
MPNIPs	Q (mg/g)	19.42	14.09	14.47	11.69
	K_d (mL/g)	17.42	12.09	12.41	9.99
	K		1.44	1.40	1.74
K'			1.81	1.86	1.53

K_d = binding amount/equilibrium concentration, $K = K_{d\,(\text{CGA})}/K_{d\,(\text{similar components})}$,

$K' = K_{MPMIPs}/K_{MPNIPs}$

Fig. S1. Wide-angle XRD patterns of Fe_3O_4@mSiO_2@MIPs (a) and MPMIPs (b).

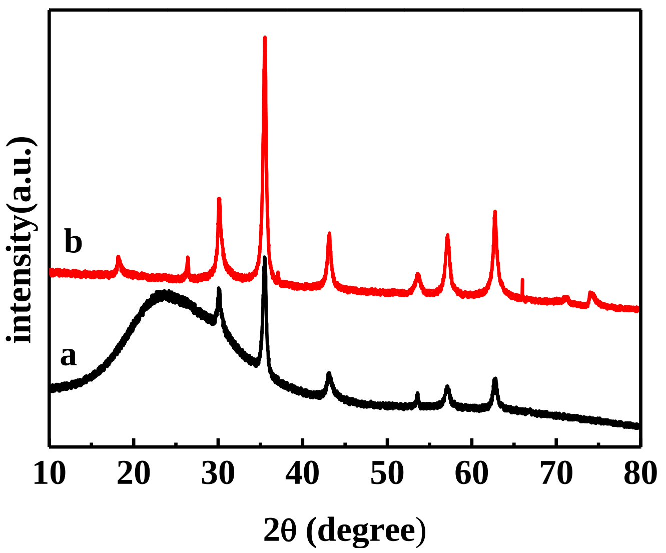

Fig. S2. TGA curve of Fe_3O_4@mSiO_2@MIPs (a), MPMIPs (b).

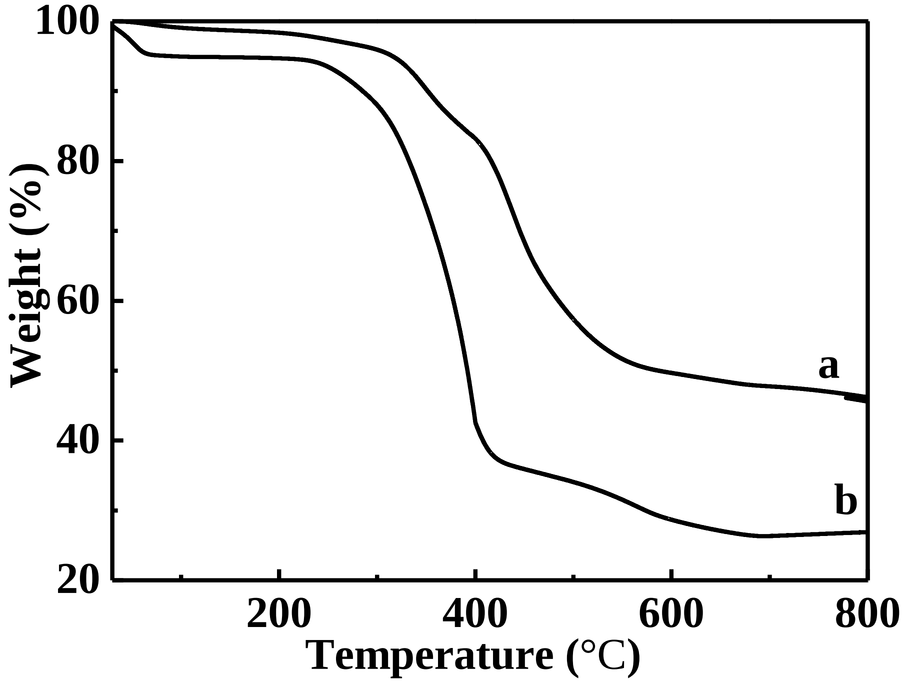

Supplement: Supplementary file 1 [file molecules-23-01554-s001.pdf]
